# Targeting IgE Antibodies by Immunoadsorption in Atopic Dermatitis

**DOI:** 10.3389/fimmu.2018.00254

**Published:** 2018-02-19

**Authors:** Michael Kasperkiewicz, Enno Schmidt, Ralf J. Ludwig, Detlef Zillikens

**Affiliations:** ^1^Department of Dermatology, University of Lübeck, Lübeck, Germany; ^2^Lübeck Institute of Experimental Dermatology, University of Lübeck, Lübeck, Germany

**Keywords:** atopic dermatitis, autoantibody, IgE, immunoadsorption, inflammation

## Abstract

One major hallmark of atopic dermatitis (AD) is the elevated level of total serum IgE, which has been reported to be partly of the autoreactive type in a subset of patients. Immunoadsorption (IA) has been successfully applied in various classical autoantibody-mediated diseases such as pemphigus. Recent reports proposed the use of IA also for patients with severe AD and high total serum IgE levels. In this mini-review, we summarize the current knowledge about this novel treatment approach for AD and briefly discuss the so far incompletely known role of autoreactive IgE as potential target of IA therapy in this common inflammatory skin disorder.

## Introduction

Atopic dermatitis (AD) is a chronic or chronically relapsing, eczematous, pruritic skin disease affecting 2–20% of the general population, which can considerably affect the patient’s quality of life. It is often associated with type I allergic diseases, including food allergy, asthma, and allergic rhinitis (1).

The pathophysiology of AD is complex and involves the interplay of genetic, immunological, and environmental factors. Both skin barrier dysfunction due to decreased filaggrin expression and a biphasic T cell-mediated immune reaction driven by a Th2 phenotype in childhood and acute disease phase that is shifted toward a Th1 signal in the chronic stage are considered to play important roles in the disease process. One major hallmark of the disease is the elevated level of total serum IgE in approximately 80% of AD patients. In fact, AD patients mount IgE antibody responses to various environmental allergens and autoantigens, which will be discussed later in this review (1).

The drug therapy of AD consists of topical emollients for barrier dysfunction and topical corticosteroids and calcineurin inhibitors for skin inflammation, as well as, in severe cases, systemic anti-inflammatory agents such as cyclosporine A. However, these conventional treatments may not show uniform efficacy and can be limited by severe side effects. Thus, several new targeting therapies, including the newly approved promising IL-4 receptor α chain antagonist dupilumab, have emerged (1).

Clinical benefit has been also achieved through sequestering of free IgE by the anti-IgE monoclonal antibody omalizumab in AD patients with poor response to traditional therapy, although some controversial results have been reported. Effects of this treatment seem to depend on baseline total serum IgE levels as less favorable clinical responses were associated with concentrations of more that 700 kU/L compared with lower levels. This observation is based on a recent systematic review and meta-analysis of 103 AD patients from 13 studies, which overall revealed that 43% of these patients could achieve an excellent clinical response after omalizumab treatment, while 27% showed satisfying results and 30% had irrelevant clinical changes or deterioration (2). Restricted clinical efficacy in some cases may be related to insufficient IgE neutralization by omalizumab, for which the recommended dosing table is limited to 150–1,200 mg/month (2, 3).

In the recent years, immunoadsorption (IA) has evolved as an alternative anti-IgE treatment for patients with severe AD displaying high total serum IgE (4–9). In addition, it has been reported that IA can increase the threshold of IgE immunoreactivity to different food allergens and thus reduce the risk of anaphylaxis in multiple food allergy (10, 11). Most recently, a pilot study indicated that IA may also be used to treat patients with allergic asthma (12). While much more experience with this treatment exists in classical autoantibody-mediated diseases such as pemphigus (13), application of IA in AD is limited to only a few centers so far. The active principle of most available adsorber types is the non-specific removal of the different immunoglobulins from the patient’s circulation (13), although an IgE-specific adsorber column has more recently become available (5, 8–12).

This mini-review summarizes the current knowledge about IA in AD and briefly discusses the role of autoreactive IgE as potential target of IA therapy in this inflammatory skin disease.

## IA in AD

### IA Protocols

To date, six studies on the use of IA in 2–50 patients per case series with severe AD and high total serum IgE levels have been published (summarized in Table 1) (4–9). The first IA treatment protocol for AD has been introduced by our group in 2011. In this pilot study, patients with treatment-refractory AD and total serum IgE levels >4,500 kU/L were treated with a total of 10 IA on days 1–5 (week 1) and days 29–33 (week 5) by using adsorption columns that contained polyclonal sheep antihuman immunoglobulin antibodies binding the different human immunoglobulins with similar affinity (referred to as panimmunoglobulin IA) (4). The same treatment protocol was used in our two following studies, with the major difference that the adsorber consisted of monoclonal mouse antihuman IgE (referred to as IgE-selective IA) (5, 9). Three other independent studies used panimmunoglobulin IA in patients with severe AD and high total serum IgE applying (i) 1–5 cycles of 5 consecutive aphereses at monthly intervals (6), (ii) 1 cycle of 2–4 consecutive aphereses followed by omalizumab every 2 weeks for 24 weeks (7), and (iii) 3 cycles of 3–4 consecutive aphereses at weeks 1, 3, and 8 (8). Some patients of the latter study were treated with IgE-selective IA (8). In all studies except for the trial combining IA with omalizumab (7), the patients’ preceding topical and systemic treatments for AD were continued during IA therapy and modified as needed (4–6, 8, 9).

**Table 1:** Summary of published studies relating to IA and AD.

Reference	Patient characteristics	IA protocol	Concomitant therapy	Follow-up time after IA start (months)	Main clinical outcomes	Main laboratory outcomes	Side effects
Kasperkiewicz et al. (4)	12 patients, 3 females, and 9 males; 24–66 (mean 42) years; SCORAD 55–98 (mean 78.6); total serum IgE 4,666–86,119 (mean 22,034) kU/L	2 cycles of 5 consecutive panimmunoglobulin IA (TheraSorb-Ig^®^, Miltenyi Biotec) at weeks 1 and 5	Topical corticosteroids/calcineurin inhibitors, oral antihistamines, and cyclosporine A	3	Mean SCORAD improvement by 38% (week 3), 46% (week 5), 56% (week 9), and 59% (week 13); parallel improvement of EASI	Temporal mean serum IgE reduction by >90% per IA cycle (similarly for IgG/IgM/IgA); sustained reduction of skin-bound IgE as well as histologic alterations (hyperkeratosis, spongiosis, acanthosis, and dermal infiltrate)	Central venous catheter-related *Staphylococcus aureus* septicemia (*n* = 1)

Kasperkiewicz et al. (5)	2 male patients; 40–60 years; SCORAD 66 and 77 (mean 71.5); total serum IgE 17,020 and 46,540, (mean 31,780) kU/L, respectively	2 cycles of 5 consecutive IgE-selective IA (TheraSorb-IgE^®^, Miltenyi Biotec) at weeks 1 and 5	Topical corticosteroids/calcineurin inhibitors, oral antihistamines, and cyclosporine A	6	Mean SCORAD improvement by 33% (week 3), 37% (week 5), 54% (week 9), 53% (week 13), 55% (week 17), and 49% (week 25)	Temporal mean serum IgE reduction by >90% per IA cycle (36–49% for IgG/IgM/IgA)	None

Daeschlein et al. (6)	7 patients, 2 females and 5 males; 17–61 (mean 35.3) years; SCORAD 21.3–77 (mean 52); total serum IgE 724–28,500 (mean 11,015) kU/L	1–5 cycles of 5 consecutive panimmunoglobulin IA (TheraSorb-Ig flex^®^, Miltenyi Biotec) at monthly intervals	Topical corticosteroids/calcineurin inhibitors, oral antihistamines, cyclosporine A	12–18	Mean SCORAD improvement by 25.1% (after 1. IA cycle), 27.9% (after 2. IA cycle), 37.6% (after 3. IA cycle), 24.1% (after 4. IA cycle), and 11.1% (after 5. IA cycle)	Temporal mean serum IgE reduction by 74–80% per IA cycle	Drop of blood pressure (*n* = 2)

Zink et al. (7)	10 patients, 2 females and 8 males; 26–65 (mean 43.7) years; SCORAD 50.2–74.6 (mean 59.9); total serum IgE 3,728–69,872 (mean 18,094) kU/L	1 cycle of 2–4 consecutive panimmunoglobulin IA (TheraSorb-Ig flex^®^, Miltenyi Biotec) followed by omalizumab every 2 weeks for 24 weeks	Topical corticosteroids	12	Mean SCORAD improvement by 27% (week 3), 40% (week 13), 55% (week 25), and 19% (re-increased; week 49); parallel improvement and re-increase of VAS subjective severity score	Mean serum IgE reduction by 58–86% after 2–4 IA, respectively; serum IgE and TARC levels decreased continuously during omalizumab therapy and re-increased during treatment-free follow-up	IA-related: dizziness (*n* = 1) and fatigue (*n* = 2); omalizumab-related: headache (*n* = 1), abdominal pain (*n* = 1), axillary lymph node swelling (*n* = 1), and elevation of liver enzymes (*n* = 3)

Reich et al. (8)	50 patients, 20 females and 30 males; 21–75 (mean 45.6) years; mean EASI and SCORAD 21.3 and 40.5, respectively; median total serum IgE 6,700 k	3 cycles of 3–4 consecutive panimmunoglobulin IA (*n* = 24; TheraSorb-Ig flex^®^, Miltenyi Biotec) or IgE-selective IA (*n* = 26; TheraSorb-IgE^®^, Miltenyi Biotec) at weeks 1, 3, and 8	Topical and systemic corticosteroids, topical calcineurin inhibitors, cyclosporine A, methotrexate, and mycophenolate mofetil	8	Median EASI improvement by 39 and 47% (week 6), 52 and 45% (week 12), and 61 and 60% (week 32) in the panimmunoglobulin and IgE-selective IA group, respectively; parallel improvement of SCORAD, POEM, and DLQI	Temporal median serum IgE reduction by 85 and 90% per IA cycle in the panimmunoglobulin and IgE-selective IA group (85 and 20% for IgG), respectively	Panimmunoglobulin IA group only: herpes labialis (*n* = 2), herpes keratitis (*n* = 1), bacterial conjunctivitis (*n* = 1), bacterial sinusitis (*n* = 1), air embolism during central venous catheter placement (*n* = 1), cubital vein thrombophlebitis (*n* = 1), and generalized cutaneous allergic drug reaction (*n* = 1)

Kasperkiewicz et al. (9)	10 patients, 3 females and 7 males; 18–70 (mean 40.3) years; SCORAD 61.5–81 (mean 67.5); total IgE 931–21,510 (mean 5,377) kU/L	2 cycles of 5 consecutive IgE-selective IA (TheraSorb-IgE^®^; Miltenyi Biotec) at weeks 1 and 5	Topical and systemic corticosteroids, topical calcineurin inhibitors, oral antihistamines	6	Mean SCORAD improvement by 19% (week 3), 29% (week 5), 43% (week 9), 21% (week 13), 25% (week 17), and 29% (week 25)	Temporal mean serum IgE reduction by > 90% per IA cycle (35–43% for IgG/IgM/IgA)	Central venous catheter-related *S. aureus* septicemia (*n* = 1), fatigue (*n* = 1), and edema of hands and feet (*n* = 1)

*AD, atopic dermatitis; DLQI, Dermatology Life Quality Index; EASI, Eczema Area and Severity Index; IA, immunoadsorption; POEM, Patient-Oriented Eczema Measure; SCORAD, Scoring Atopic Dermatitis; TARC, thymus and activation regulated chemokine; VAS, Visual Analog Scale*.

### Effects on the Clinical Course

During a follow-up time of 3–18 months, the published studies uniformly revealed a satisfactory initial linear decrease of the disease severity [measured by Scoring Atopic Dermatitis (SCORAD) or Eczema Area and Severity Index] as early as within the first 3 weeks after initiation of IA regardless of the IA treatment protocol used (4–9). Results from our three reports indicate that IA treatment resulted in continuous and stable SCORAD improvements of up to approximately 60% for at least 3–6 months (4, 5, 9), although some minor re-increases in the SCORAD (being still lower than before initiation of IA) were observed in patients treated by IgE-selective IA during the second half of the observation period (5, 9). In the study by Reich et al., clinical effects remained widely stable until 6 months of follow-up with no major differences in clinical efficacy between panimmunoglobulin IA- and IgE-selective IA-treated patients (8). Combined use of IA and omalizumab resulted in a steady decrease of the SCORAD throughout the 6-month treatment period, whereas a reverse trend was observed during treatment-free follow-up of another 6 months (7). No additional benefit with regard to further SCORAD improvement was observed in one study when more than three monthly cycles of five consecutive panimmunoglobulin IA were applied, although one patient who completed five IA cycles exhibited a long lasting clinical benefit over 12 months (6).

Of note, the studies demonstrated that IA was associated with disease amelioration in patients’ refractory to multiple conventional treatments including cyclosporine A, which could be partly reduced or discontinued after IA (4–6, 8, 9).

### Effects on Circulating IgE and Other Serum Parameters

Results from our three reports revealed that serum IgE levels were effectively reduced by a mean of more than 90% with each IA cycle regardless of the specificity of the adsorber (4, 5, 9). Similar IgE reductions were observed in the other three studies ranging from an average of approximately 60–90% depending on the length of the IA cycle (6–8). In contrast to panimmunoglobulin IA, however, which reduced other immunoglobulin isotypes to a similar degree (4, 8), serum concentrations of IgM, IgA, and IgG were decreased by only less than half using IgE-selective IA (5, 8, 9). The concomitant decrease of immunoglobulins other than IgE by the IgE-selective adsorber is considered to be non-specific and explained by IA-related elution and dilution procedures (5, 8, 9). Effects of IgE-selective and –non-selective IA on peripheral levels of IgE and other immunoglobulins were transient since immunoglobulin levels started to rise again following each IA procedure and precycle values remained widely similar over time (4–6, 8, 9). Thus, considering the generally steady decrease of the disease activity following IA, total serum IgE levels only incompletely paralleled the clinical course of the patients. By contrast, a different observation was made by the combinatory use of IA and omalizumab. After initial reduction in total circulating IgE by IA, free serum IgE levels continued to fall during omalizumab treatment [in parallel with SCORAD improvement and reduction in serum levels of the biomarker thymus and activation regulated chemokine (TARC)] and started to increase again (in parallel with SCORAD worsening and increase of TARC) during follow-up after anti-IgE therapy was discontinued (7).

### Effects on Skin-bound IgE and Other Skin Parameters

In contrast to the abovementioned kinetics of circulating IgE, a continuous reduction of the amount of skin-bound IgE was observed by immunohistochemistry in our initial IA pilot study (4). It can be hypothesized that a redistribution of tissue-bound IgE from inflammatory skin sites into the intravascular compartment occurs, which may at least partly explain the rebound phenomenon of serum IgE levels following IA. In addition, repetitive skin biopsies after IA revealed a steady decrease in skin infiltration by antigen-presenting cells and T cell as well as improvement of altered epidermal morphology including hyperkeratosis, spongiosis, and acanthosis (4). In this context, it is worth noting that omalizumab has been previously shown to inhibit antigen processing and presentation to T cells by downregulating the expression of the high-affinity IgE receptor FcepsilonRI on dendritic cells (14). Thus, impairment of IgE-mediated antigen exposure in the skin could represent a possible mechanism of action of IA contributing to disease amelioration in AD patients.

### Safety

IA in patients with AD, who usually have an increased genetic susceptibility to skin infections by pathogens such as *Staphylococcus aureus* and *Herpes simplex* virus (1), is generally well tolerated (4–9). The selective IgE adsorber has been developed to decrease the potential higher risk of infections due to the parallel considerable reduction of protective immunoglobulins as it occurs with panimmunoglobulin IA. In fact, in the study by Reich et al., infectious adverse events, including bacterial conjunctivitis/sinusitis and herpes labialis/keratitis, were observed in AD patients treated by panimmunoglobulin IA but not IgE-selective IA (8). By contrast, however, we reported two cases of *S. aureus* septicemia, one occurring after panimmunoglobulin IA and the other following IgE-selective IA, both performed by a central venous catheter (4, 9). Thus, IgE-selective IA using peripheral venous access seems most preferable, although further studies are needed to better define the safety profile of this treatment in AD patients.

### Autoreactive IgE as Potential Target of IA in AD

With the help of Th2 cytokines, activated B cells undergo production of IgE antibodies, which have been reported to be partly of the autoreactive type in AD (15–18). More than 140 autoantigens triggering IgE autoantibodies in AD patients have been reported (16), some of them potentially binding to cell membrane and intracellular structures of cultured human keratinocytes (15). IgE autoantibodies can either be specific for autoantigens (Homs 1–5, antinuclear antibodies, DSF70, and p80-coilin) or cross-react with environmental allergens due to their high homology with self-antigens (manganese superoxide dismutase, thioredoxin, profilin, and acidic ribosomal P2 protein) (18). In fact, a systematic review involving 1,253 AD patients and 1,391 control subjects found autoreactivity in up to 91% compared with 0–12%, respectively, although it remains widely unknown whether this is a cause or consequence of skin inflammation in AD (17).

It has been hypothesized that scratching-induced tissue damage can lead to the release of autoantigens that bind to IgE autoantibodies. This may consecutively result in immediate-type hypersensitivity reactions as well as T cell activation and cytokine secretion through IgE cross-linking on the cell surface and IgE-mediated autoantigen presentation, respectively (18). In contrast to other IgE-driven diseases, such as bullous pemphigoid (19), however, it is less known whether autoreactive IgE in AD patients are truly pathogenic or only represent a bystander epiphenomenon.

Pathogenicity of IgE-mediated autoreactivity in AD is supported by the following reported evidence: (i) IgE autoantibodies are not present in other allergic diseases, such as allergic asthma or allergic rhinoconjunctivitis, which are characterized by increased total IgE levels (17); (ii) existence of pro-inflammatory cytokine-producing autoreactive T cells specific for autoantigens [e.g., α-NAC (Homs 2)] for which autoreactivity has been described on the humoral level (20, 21); (iii) disease severity in AD patients is higher in autoreactive patients than in non-autoreactive ones (15, 17); (iv) SCORAD values and serum TARC levels correlate with IgE autoantibodies to human manganese superoxide dismutase and DSF70, respectively (22, 23); and (v) cyclosporine A-induced improvement of skin symptoms in AD patients is accompanied by reduction in IgE autoreactivity (but not IgE antibodies to exogenous allergens), with a reverse trend without treatment; clinical improvement precedes decrease of IgE autoreactivity, potentially suggesting reduced tissue injury-driven exposure of self-antigens to autoreactive B cells in these patients (24, 25).

Therefore, it is tempting to speculate that disease amelioration by IA in some AD patients may also, at least in part, be attributed to a decrease of IgE autoreactivity.

## Conclusion

Clinical evidence suggests that IA seems to be an effective treatment option for patients severely affected by AD with highly elevated IgE serum levels. IA is associated with temporal and sustained reduction of circulating and skin-bound IgE, but the exact mechanisms underlying the clinical response in AD patients remain to be elucidated (4–9). Although the role of IgE in AD is still a matter of debate, these findings favor a pathogenic potential of IgE antibodies in this disorder. Future studies should explore whether a particular subset of AD patients who have circulating autoreactive IgE antibodies may especially benefit from this type of direct antibody depletion therapy.

## Author Contributions

MK wrote the paper with the help of ES, RL, and DZ.

## Conflict of Interest Statement

All authors have a research cooperation with Miltenyi Biotec. The authors declare that the research was conducted in the absence of any commercial or financial relationships that could be construed as a potential conflict of interest.
